# Effect of founder breeds on genotype imputation accuracy in Canchim cattle

**DOI:** 10.1007/s13353-026-01060-z

**Published:** 2026-04-16

**Authors:** Roney Teixeira, Maria Victória Henrique Genuíno, Ayrton Fernandes de Oliveira Bessa, Giovanna Maria dos Santos Câmara, Ana Carolina de Jesus Oliveira, Josineudson Augusto II de Vasconcelos Silva, Lucio Flavio Macedo Mota, Fernando Baldi, Luciana Correia de Almeida Regitano, Cintia Righetti Marcondes, Priscila Arrigucci Bernardes, Donagh Pearse Berry, Danísio Prado Munari, Marcos Eli Buzanskas

**Affiliations:** 1 Universidade Federal da Paraíba (UFPB), Areia, PB 58397-000 Brazil; 2 School of Agricultural and Veterinarian Sciences (FCAV), São Paulo State University (UNESP), Jaboticabal, SP 14884-900 Brazil; 3 School of Veterinary Medicine and Animal Science (FMVZ), São Paulo State University (UNESP), Botucatu, SP 18618-681 Brazil; 4 Faculdade de Zootecnia e Engenharia de Alimentos (FZEA), Universidade de São Paulo (USP), Pirassununga, SP 13635-900 Brazil; 5 Embrapa Southeast Livestock, São Carlos, SP 13560-970 Brazil; 6 Departamento de Zootecnia e Desenvolvimento Rural, Universidade Federal de Santa Catarina, Florianópolis, SC 88034-000 Brazil; 7 Institute for Future Farming Systems, School of Health, Medical & Applied Sciences, CQ University, North Rockhampton, QLD 4701 Australia; 8 Teagasc, Animal and Grassland Research and Innovation Centre, Fermoy Co, Cork , Moorepark P61C997, Ireland

**Keywords:** Animal breeding, Crossbred, Genomic selection, Genotypes

## Abstract

Genotype imputation is a technique used to infer unobserved genotypes based on reference panels, allowing increased marker density and cost-effective optimization for genomic selection. This study aimed to evaluate whether the inclusion of genotypes from the founder breeds Nelore (NE) and Charolais (CH) improves the imputation accuracy in the composite beef cattle breed Canchim (CA). The populations studied consisted of 804 NE, 897 CH, and 392 CA animals, all genotyped using high-density panels (777,962 SNP – single nucleotide polymorphisms). CA animals had their genotypes masked to simulate a medium-density panel (54,609 SNP). Fourteen imputation scenarios were evaluated, varying according to breed, sex, year of birth, and lineage. Imputation accuracy was determined based on the percentage of correctly imputed genotypes (PERC) and the squared Pearson’s correlation between observed and imputed genotypes (R^2^). PERC values ranged from 66.52% to 97.39% and R² from 0.6352 to 0.9780. The scenarios that included NE, CH, and CA (males or animals born before 2004) as the reference population for imputing CA females or CA animals born after 2004 showed the highest imputation accuracies. Therefore, the use of founder breeds in the reference population improves the accuracy of genotype imputation in CA cattle. The results indicate that a multibreed reference population, incorporating founder breeds, could provide a more robust and informative genetic basis for imputing composite cattle.

**Supplementary Information:**

The online version contains supplementary material available at https://doi.org/10.1007/s13353-026-01060-z.

## Introduction

Brazilian beef cattle farming has continuously invested in technologies to optimize productivity, feed efficiency, and meat quality, thereby consolidating the country as one of the world’s leading beef exporters (Senna Carvalho et al. 2023). Among the strategies adopted, crossbreeding has enabled the development of composite breeds adapted to tropical conditions, such as the Canchim cattle. Created in the 1940s, this breed combines the productive and meat quality traits of European cattle with the adaptability and parasite resistance of indicine cattle. Canchim animals typically have a genetic composition of 5/8 Charolais (*Bos taurus taurus*) and 3/8 Nelore (*Bos taurus indicus*), although variations exist depending on the breeding scheme (Alencar 1988).

Canchim is considered a genetic alternative for beef production systems, especially in tropical and subtropical regions, due to its feed efficiency, high growth rate, good feed conversion, and superior meat quality, with marbling and tenderness suited to consumer market demands (Santiago et al. 2017). Despite its relevance, the Canchim herd in Brazil remains relatively small when compared to other commercial breeds (Duarte et al. 2022). Four mating schemes are used to obtain Canchim cattle, three of which are referred to as the old lineage and one as the new lineage, as described by Bessa et al. (2025). The new lineage includes polled Charolais cattle from the United States, Argentina, and England in its crosses, whereas the old lineage includes Charolais cattle mainly of French origin (Marcondes 2018).

Selection in composite breeds generally follows methods used in purebreds (Gregory et al. 1995). However, the genome of these breeds contains fragments of the original ancestral genomes in blocks of varying sizes resulting from gene recombination events (Pugach et al. 2011). It therefore remains unclear whether the composition of the founding breeds is stable after crossbreeding. This knowledge is necessary to determine the future use and management of composite cattle breeds (Paim et al. 2020).

Advances in genomic selection and genome-wide association studies have improved selection accuracy and helped identify causal variants underlying traits of economic interest (Buzanskas et al., 2017a; Meuwissen et al. 2016). These studies rely on single nucleotide polymorphisms (SNP) panels of varying densities. Low-density panels are cheaper but may lack key markers across populations. Imputation addresses this limitation by inferring missing genotypes from reference populations genotyped at higher densities, leveraging linkage disequilibrium and shared haplotypes to improve cost-effectiveness (Wang et al. 2016).

In beef cattle, imputation from low- to medium-density panels has yielded similar prediction accuracies to direct medium-density genotyping, though accuracy in crossbreds was lower than in purebreds (Wang et al. 2016). Ventura et al. (2014) further showed that including all founder breeds in the reference population enhances imputation performance in composite cattle. Thus, this study aimed to evaluate the accuracy of imputation from medium- to high-density genotypes in Canchim cattle, considering different scenarios that included Nelore and Charolais founder genotypes in the reference populations.

## Materials and methods

Genotypes of 392 Canchim (CA) animals were considered for the target population. A total of 280 and 112 CA animals were from the new and old lineages, respectively. These animals were raised on pasture with mineral supplementation throughout the year and were progeny of 49 sires and 355 dams, originating from seven herds located in two Brazilian states (São Paulo and Goiás). The main reference population included 804 Nelore (NE) and 897 Charolais (CH) animals. Nelore animals were raised in feedlots located in the Brazilian states of São Paulo and Mato Grosso do Sul, which present tropical, warm, and rainy climate. Charolais animals were from Ireland, in which beef production relies on in situ grazed perennial ryegrass pastures and the country has a temperate climate.

All animals were genotyped with the high-density Illumina BovineHD Genotyping BeadChip (777,962 SNP) and considered the ARS-UCD 1.2 reference map (Rosen et al. 2020). Quality control (QC) was performed in PLINK v.1.9 (Purcell et al. 2007), considering only autosomal markers. SNP and samples with call rate < 95% and SNP with MAF < 1% were excluded. After merging datasets from CA, NE, and CH, QC was applied, and then data from each breed were separated to construct imputation scenarios. After the QC, a total of 2,093 animals, 41,474 SNP (50k panel), and 700,992 SNP (HD panel) were considered for all imputation scenarios.

Fourteen imputation scenarios were tested (Table 1). In S1–S3, all CA animals were the target population, while in S4–S10 part of them were also in the reference population. In S11–S14, all CA from the old lineage were considered in the reference population. NE and CH were used as reference, either separately or combined. Reference animals retained high-density genotypes, while imputation animals had their HD panel reduced to BovineSNP50 BeadChip (50 K).

These scenarios were developed considering practical aspects, where young animals and/or females are genotyped with lower-density panels, which represent lower costs, while older animals, with high representation in the population and high accuracies for genetic evaluations, are genotyped with high-density panels.

Imputation was carried out with FImpute v.3 (Sargolzaei et al. 2014), which identifies haplotypes based on linkage disequilibrium, reflecting close or distant kinship. Population stratification in each scenario was assessed by principal component analysis in PLINK v.1.9 (Purcell et al. 2007). Genetic divergence between reference and target populations was measured with the fixation index (Fst) of Weir and Cockerham (1984). Imputation accuracy was evaluated by the percentage of correctly imputed genotypes (PERC) and the squared Pearson’s correlation between observed and imputed genotypes (R²), comparing imputed markers with the original HD panel.

## Results and Discussion

The comparison of imputed markers with those present in the original HD panel (Table 1) revealed a PERC ranging from 66.52% (S1) to 97.39% (S6) and R² varied from 0.6352 (S1) to 0.9780 (S6). When considering only the founders breed as reference populations, the imputation accuracy showed a wider variation with PERC from 66.52% to 90.07% and R² from 0.6352 to 0.9121. On the other hand, all the other scenarios (S4 to S14), which considered CA animals in both the imputation and reference populations, exhibited PERC varying from 89.79% (S14) to 97.39% (S6) and R² from 0.9061 (S14) to 0.9780 (S6). The Fst values (Supplementary material - Figure S1) ranged from 0.0025 (S7) to 0.1264 (S4). Principal components (PC) 1 and 2 (Supplementary Figure S1) capture approximately 55% to 97% of the total variance in the data. In Supplementary Figure S2, the imputation accuracies for animals are presented.

In scenarios S4 to S14, the PC analyses showed overlap between the imputation and reference populations, indicating greater genetic proximity among individuals, which explains the higher accuracies observed. In contrast, S1 and S2 revealed a clear separation between the founder populations (NE and CH) and the CA breed, suggesting lower haplotype sharing and, consequently, lower imputation accuracy. S3 showed an intermediate distance between breeds, with CH animals positioned closer to CA individuals, reflecting their greater genetic contribution to the formation of the breed. Similar results were reported by Duarte et al. (2022), who also identified genetic proximity between CA and CH animals through PC analysis.

Additionally, Duarte et al. (2022) observed a low degree of genetic divergence between these two breeds, as indicated by an Fst value of 0.10. Between CA and NE, however, these authors reported an average Fst of 0.24, indicating greater genetic differentiation between these breeds. These results reinforce that genetic similarity between reference and target populations is a factor in determining imputation accuracy, as the use of an adequately representative reference population enables a broader capture of the genetic diversity of the target population (Heidaritabar et al. 2015).

Due to the presence of different Canchim lineages in this study, obtained through four mating schemes (Bessa et al. 2025), the formation of clusters in the PC analyses of scenarios S1, S4, S7, S8, S11, S12, S13, and S14 can be observed. According to Buzanskas et al. (2017b), these schemes produce Canchim animals with distinct Charolais-Nelore breed proportions, which may reflect the genetic variability present in the breed.

Supplementary Figure S2 depicts the distribution of accuracies per animal for each imputation scenario. We highlight the presence of outliers, mainly in S1, S2, and S3, indicating that these scenarios poorly imputed the missing genotypes. In general, the minimum and maximum R² and PERC values per animal were 0.5571 (S1) and 0.9941 (S7), and 61.12% (S1) and 99.29% (S7), respectively. Specifically for scenario S10, the minimum and maximum values for R² and PERC were 0.9196 and 0.9935, and 90.74% and 99.22%, respectively, representing the smallest ranges (0.0739 and 8.48%) among the scenarios evaluated.

Considering the size of the reference population in the studied scenarios, it can be observed that although scenarios S1, S2, and S3 have a larger reference population compared to the target population, low genetic proximity existed, and it may have resulted in lower imputation accuracies. However, we can highlight that the inclusion of NE + CH animals in the reference population (S3) provided an increase in imputation accuracy for CA animals. In the other scenarios (S4-S14), although the size and breeds of the reference population varied, genotypes of CA animals were included and, therefore, higher imputation accuracies were observed.

Lower imputation accuracy was observed in scenario S1 (PERC = 66.52% and R² = 0.6096), which used exclusively NE animals as the reference population for imputing all CA animals. This result can be attributed to the lower genetic similarity between the populations, as demonstrated by the higher Fst value (0.1169, Figure S1). Conversely, scenario S2, which employed only CH animals in the reference population, achieved superior accuracy levels (PERC = 71.80% and R² = 0.7181) when compared to S1. This performance could be associated with a considerably lower Fst value (0.0385) between CA and CH populations. Therefore, the greater genetic similarity between CA and CH breeds, when compared to NE, resulted in better imputation effectiveness, that may be caused by the genetic contribution of CH in the CA genetic composition.

**Table 1:** Imputation accuracies by different scenarios in Canchim cattle

Populations		Scenarios	Number of animals		Imputation accuracy	
Target	Reference		Target	Reference	PERC (%)	R²
All CA	NE	S1	392	804	66.52	0.6352
	CH	S2	392	897	71.80	0.7181
	NE + CH	S3	392	1,701	90.07	0.9121
CA♀	NE + CA♂	S4	202	994	96.61	0.9710
	CH + CA♂	S5	202	1,087	96.34	0.9690
	NE + CH + CA♂	S6	202	1,891	97.39	0.9780
CA > 2004	CA ≤ 2004	S7	141	251	95.37	0.9601
	NE + CA ≤ 2004	S8	141	1,055	96.26	0.9677
	CH + CA ≤ 2004	S9	141	1,148	96.33	0.9688
	NE + CH + CA ≤ 2004	S10	141	1,952	97.37	0.9777
New CA lineage	Old CA lineage	S11	112	280	89.79	0.9061
	NE + Old CA lineage	S12	112	1084	92.02	0.9266
	CH + Old CA lineage	S13	112	1177	92.40	0.9321
	NE + CH + Old CA lineage	S14	112	1981	95.12	0.9564

CA = Canchim; CH = Charolais; NE = Nelore; ♂ = males; ♀ = females; > 2004 = Canchim born after 2004; ≤ 2004 = Canchim born in 2004 or before 2004; PERC = percentage of correctly imputed genotypes; (PERC); R^2^ = squared Pearson’s correlation between observed and imputed genotypes

Our results are consistent with previous studies, which consistently indicates that low imputation accuracies can be attributed to genetic divergences between the involved breeds, the pattern of linkage disequilibrium between markers, and the size of the reference population relative to the target population (Heidaritabar et al. 2016). The dependence of imputation accuracy on the genetic divergence between animals in the reference population and those in the target population is also corroborated by Bolormaa et al. (2015). In addition to genetic composition, Butty et al. (2019) showed that the size of the reference population also influences imputation accuracy, since larger populations may have greater haplotype diversity and better representation of allelic combinations, increasing the probability of correctly inferring missing genotypes in the target population. Takeda et al. (2021) demonstrate that small reference populations harbor fewer haplotypes, which can compromise imputation accuracy, especially in regions with low allele frequencies.

Scenario S3, which simultaneously considers NE and CH into the reference population, resulted in a notable increase in imputation accuracy (PERC = 90.07% and R² = 0.9121) when compared to scenarios S1 and S2. In this context, the contribution of the NE breed, although genetically more distant, still optimizes the imputation process. In scenarios S5, S9, and S13, the inclusion of CH and CA (males, older animals, or old lineage) in the reference population had a small beneficial effect on imputation accuracy when compared to scenarios S4, S8, and S12.

Scenarios S3, S6, S10, and S14 simultaneously included the founder breeds (NE and CH) in the reference population. The observed PERCs in each of these scenarios were 90.07%, 97.39%, 97.37%, and 95.12%, respectively, while R² values were 0.9121, 0.9780, 0.9777, and 0.9564, respectively. Higher accuracies were observed in S6 and S10 when compared to those in which the founder breeds were used separately (S1 and S2) and all other scenarios.

When considering male CA animals (S4, S5, and S6) or animals born before 2004 (S8, S9, and S10), together with NE and CH animals, as reference populations, higher accuracies were observed compared to scenarios that considered the old lineage (S12, S13, and S14) as reference population. It can be hypothesized that the genetic variability present in animals of different lineages may bring benefits to the imputation accuracy.

A previous study carried out by Buzanskas et al. (2021) with similar dataset, demonstrated that a larger number of haplotype blocks were shared between CA and CH animals compared to the haplotype blocks shared between CA and NE animals. These authors also found a higher frequency of long (> 500 kb) and short (< 50 kb) haplotypes shared between CA and CH animals, while between CA and NE long haplotypes were also identified, but at lower frequencies. According to Sargolzaei et al. (2014), the algorithm present in FImpute software uses family imputation if pedigree data is available (which was not considered in our study) and then exploits the population relationships by searching for long haplotype matches in the reference population, continuing the process by using short haplotypes that are related to more distant relatives. For these reasons, the scenarios that included the two founding breeds of Canchim were those that presented the greatest accuracy.

Pausch et al. (2017) found that the usage of a multibreed reference population on imputation yielded the accuracy gain for Fleckvieh and Holstein animals. According to the authors, the use of multibreed reference populations may be fundamental for the imputation of genotypes in populations with a reduced number of individuals. The scenarios evaluated by Oliveira Júnior et al. (2017) in Girolando cattle (a composite of Gyr and Holstein) are consistent with our findings when imputing from 50k to HD panels. In their study, the highest imputation accuracies were obtained when Girolando animals were included in the reference population (R² from 0.96 to 0.97), whereas substantially lower accuracies were observed when only Gyr (R² = 0.51) or, to a lesser extent, Holstein (R² = 0.75) were used. High accuracies were also achieved when multibreed reference populations were considered (R² from 0.94 to 0.97).

## Conclusion

The use of founder breeds in the reference population improves the accuracy of genotype imputation in CA cattle. Our results indicate that a multibreed reference population, incorporating the founder breeds, could provide a robust and informative genetic basis for imputing composite cattle.

## Supplementary Information

Below is the link to the electronic supplementary material.


Supplementary Material 1 (DOCX 3.33 MB)


## Data Availability

The genotypes can be available upon reasonable request.
