# Supplementary material for: Effect of founder breeds on genotype imputation accuracy in Canchim cattle
Source: J Appl Genet. 2026 Apr 16;67(3):805–10. doi: 10.1007/s13353-026-01060-z (PMC13534181; doi:10.1007/s13353-026-01060-z)
Supplement: Supplementary file 1 [file 13353_2026_1060_MOESM1_ESM.docx]

**Figure S1.** Allelic fixation index (Fst) and principal component (PC) 1 (x-axis) and 2 (y-axis) for imputation scenarios S1 to S14.

**Figure S2.** Scatter plot (A and C) and boxplot (B and D) for genotype imputation accuracy per animal in each scenario (S1 to S14) considering the squared Pearson’s correlation between observed and imputed genotypes (R²) and the percentage of correctly imputed genotypes (PERC).
